# Optical Properties of Plasmonic Mirror-Image Nanoepsilon

**DOI:** 10.1186/s11671-016-1549-8

**Published:** 2016-07-12

**Authors:** Jia-Yu Lin, Chia-Yang Tsai, Pin-Tso Lin, Tse-En Hsu, Chi-Fan Hsiao, Po-Tsung Lee

**Affiliations:** Department of Photonics and Institute of Electro-Optical Engineering, National Chiao Tung University, CPT Building, 1001 Ta-Hsueh Road, Hsinchu, 300 Taiwan

**Keywords:** Localized surface plasmon resonance, Near-field enhancement, Nanorod dimer, Nanoring, Plasmonics, Nanostructures

## Abstract

We propose a novel mirror-image nanoepsilon (MINE) structure to achieve highly localized and enhanced near field at its gap and systematically investigate its plasmonic behaviors. The MINE can be regarded as a combination of two fundamental plasmonic nanostructures: a nanorod dimer and nanoring. By adapting a nanoring surrounding a nanorod dimer structure, the nanorod is regarded as a bridge pulling the charges from the nanoring to the nanorod, which induces stronger plasmon coupling in the gap to boost local near-field enhancement. Two resonance peaks are identified as the symmetric and anti-symmetric modes according to the symmetries of the charge distributions on the ring and rod dimer in the MINE. The symmetric mode in the MINE structure is preferred because its charge distribution leads to stronger near-field enhancement with a concentrated distribution around the gap. In addition, we investigate the influence of geometry on the optical properties of MINE structures by performing experiments and simulations. These results indicate that the MINE possesses highly tunable optical properties and that significant near-field enhancement at the gap region and rod tips can be realized by the gap and lightning-rod effects. The results improve understanding of such complex systems, and it is expected to guide and facilitate the design of optimum MINE structures for various plasmonic applications.

## Background

Metallic nanostructures with localized surface plasmon resonance (LSPR) provide a method for manipulating light at the nanoscale beyond the optical diffraction limit [1, 2]. The collective oscillation of free electrons on the metallic nanostructure can be excited by incident light at specific frequencies with some unique optical phenomena, such as the subwavelength localization of electromagnetic energy, the directional scattering of light out of the nanostructure, and the formation of high-intensity hot spots at the structure surface [3]. The unique optical phenomena of metallic nanostructures have been extensively applied to various fields, including vibrational spectroscopies (surface-enhanced Raman spectroscopy and surface-enhanced infrared absorption) [4–6], plasmonic solar cells [7, 8], nanomedicine [9–11], enhanced single-emitter fluorescence [12, 13], gas detections [14–16], and optical tweezers [17–19]. However, to promote their performance, these applications require a highly localized and enhanced field, which depends on the plasmonic modes that dominate the optical properties of the nanostructure, while the plasmonic modes are quite sensitive to the geometry of nanostructure [20–22]. Nanostructures have been explored with various shapes, such as nanodisks [23], nanostars [24], nanotriangles [25], and nanospheres [26]. In addition, plasmonic resonances can be tuned and positioned within a large wavelength range from ultraviolet to mid-infrared wavelengths by adjusting the structural aspect ratio using hybrid nanostructures, such as nanoshells [27], nanorice [28], and nanorings [29]. Among these hybrid nanostructures, nanorings are particularly notable as the nanoring possesses a highly tunable plasmon resonance, uniformly enhanced field distribution in the cavity, large surface-to-volume ratio, and an open-cavity structure where the analyte could be easily accessed. These characteristics make this ring-shaped structure particularly suitable for developing biosensors [30].

The field intensity can be dramatically enhanced in the gap region of the metallic dimer due to the near-field coupling effect when two nanostructures are sufficiently close to each other. Various plasmonic dimers have been investigated, including nanosphere dimers [31], nanodisk dimers [32], and nanorod dimers [33]. In particular, nanorod dimers provide a strongly coupled mode to achieve a large field enhancement in the gap because the rod-shaped structure possesses a sharper shape that allows more charges to be accumulated at the rod tip, i.e., the lightning-rod effect [34]. In other words, the optical field can be improved by several orders of magnitude by modifying the gap distance or the rod width of a nanorod dimer. Such highly localized and enhanced near fields in the dimer system have been utilized to miniaturize the trapping spot size and simultaneously promote the induced optical forces for optical tweezers. For instance, Zhang et al. have demonstrated that 10-nm metal nanoparticles can be trapped and sensed with the nanorod dimer [35].

Combining the features of a nanoring and a nanorod dimer, we propose a novel mirror-image nanoepsilon (MINE), which is equal to two mirrored nanoscale ϵ-shaped structures, to boost the local near-field enhancement for various applications that require highly localized and enhanced field. Taking advantage of the nanorod dimer which can gain a high local field at the gap of the structure via lightning-rod and gap effects, an auxiliary nanoring structure is adopted surrounding the nanorod dimer to form the MINE structure for further field enhancement. The nanoring can first induce and support excess charges, and then the nanorod functions as a bridge to pull these excess charges from the nanoring to the nanorod, inducing stronger plasmon coupling to provide a larger near field around the gap. The MINE structure is fabricated and simulated to explore its plasmonic behaviors. Two resonance peaks corresponding to symmetric and anti-symmetric modes are identified. The symmetric mode is preferred because its charge distribution can support a stronger local near field with a concentrated distribution around the gap. We then investigate the influence of geometry on the optical properties of MINE structure for obtaining a highly localized and enhanced near field.

## Methods

### Fabrication

**Fig. 1:**
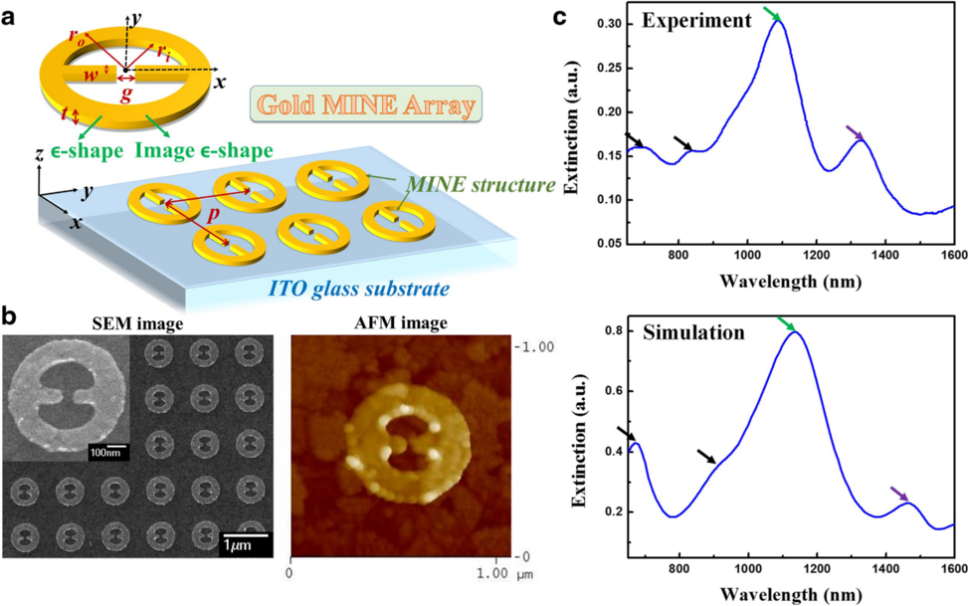
**a** Scheme of the MINE array design. **b** SEM and AFM images of the fabricated gold MINE array with *t* = 31 nm, *g* = 51 nm, *w* = 77 nm, *r*_*i*_ = 174 nm, *r*_*o*_ = 304 nm, and *p* = 1000 nm. The *inset* shows a magnified SEM image of a single gold MINE structure. **c** Extinction spectra of the MINE structure from the experiment and simulation

A schematic illustration of the gold MINE array with structural thickness *t*, gap *g*, rod width *w*, inner radius *r*_*i*_, outer radius *r*_*o*_, and period *p* is shown in Fig. 1a. The coordinate origin is set at the center of the gap on the surface of the substrate. Gold MINE arrays were manufactured on a commercial indium tin oxide (ITO) glass substrate that eliminates charge accumulation during electron beam lithography (EBL). First, the ITO glass substrate was cleaned by consecutive 5-min sonication cycles in acetone, isopropyl alcohol, and deionized water and subsequently dried using N_2_. The substrate was then spin-coated with a 150-nm electron-beam-resist polymethylmethacrylate (PMMA) layer, and the MINE arrays with dimensions of 60 × 60 μm^2^ were defined on the PMMA layer by EBL. After the development process, the substrate was deposited a 30-nm gold thin film using thermal evaporation. Finally, a lift-off procedure was implemented by immersing the sample into acetone for 48 h in an ultrasonic oscillator. The sample was subsequently put into a stripper at 45 °C for 10 min in an ultrasonic oscillator to remove the residual PMMA. The morphology and dimension of the fabricated MINE structure were measured via scanning electron microscope (SEM) and atomic force microscope (AFM).

### Optical Measurement

The extinction spectra of the gold MINE arrays were measured using a homemade horizontal transmission spectroscopy with a halogen lamp as a light source. The optical setup for the transmission spectrum measurement can be separated into three main components, including the front focusing system, the back focusing system, and an optical spectrum analyzer. The front and back focusing systems ensured that the focal planes of the 20× objective lens were situated at the MINE array, and the visible charge-coupled device (CCD) and television screen were utilized to display the focused image of the MINE structures. The halogen lamp served as the incident light source that passed through an aperture to form collimated light. The polarization of the incident light was parallel to the major axis of the rod dimer in the MINE structure. Then, the light was focused on the gold MINE arrays through a 20× objective lens, and the spot size was approximately 60 μm in diameter. Finally, the transmitted light was collected by another 20× objective lens and fed into a multimode glass fiber connected to the optical spectrum analyzer. The extinction spectrum was calculated by − log [*I*_out_(*λ*)/*I*_ref_(*λ*)], where *I*_out_(*λ*) and *I*_ref_(*λ*) are the intensities of transmitted light with and without the MINE arrays, respectively.

## Results and Discussion

### LSPR Characteristic of MINE Structure

Figure 1b shows SEM and AFM images of the fabricated gold MINE array with average *t* = 31 nm, *g* = 51 nm, *w* = 77 nm, *r*_*i*_ = 174 nm, *r*_*o*_ = 304 nm, and *p* = 1000 nm. The inset in the SEM image shows a single MINE structure at high magnification. No apparent electron-beam-resist PMMA residue remains on the surrounding surfaces or in the cavities of the nanostructures. The three-dimensional finite element method (3D FEM), processed via COMSOL Multiphysics software, is implemented to investigate the optical properties of the MINE structure. In our simulation, the dielectric function of the gold MINE structure is described by the Drude-Lorenz model [36]. The environmental refractive index is set as 1.0 for air, and dispersion of the ITO substrate is considered [37]. The scattering boundary condition is applied for all external boundaries of the computational domain to investigate the optical behavior without considering any optical couplings. All simulation results come from a single MINE structure. The dimensions of the simulated structure with *t* = 30 nm, *g* = 50 nm, *w* = 75 nm, *r*_*i*_ = 175 nm, and *r*_*o*_ = 305 nm are chosen to match the fabricated dimensions. The incident optical field (from the top) with amplitude of 1 V/m is polarized in *x*-direction to excite LSPR in the central gap. In addition, considering fabrication feasibility, the right-angle apexes of the nanorod were curved with a radius of curvature of 10 nm. The measured and simulated extinction spectra are provided in Fig. 1c. Two main resonance peaks (green and purple arrows) occur in the near-infrared (NIR) region.

**Fig. 2:**
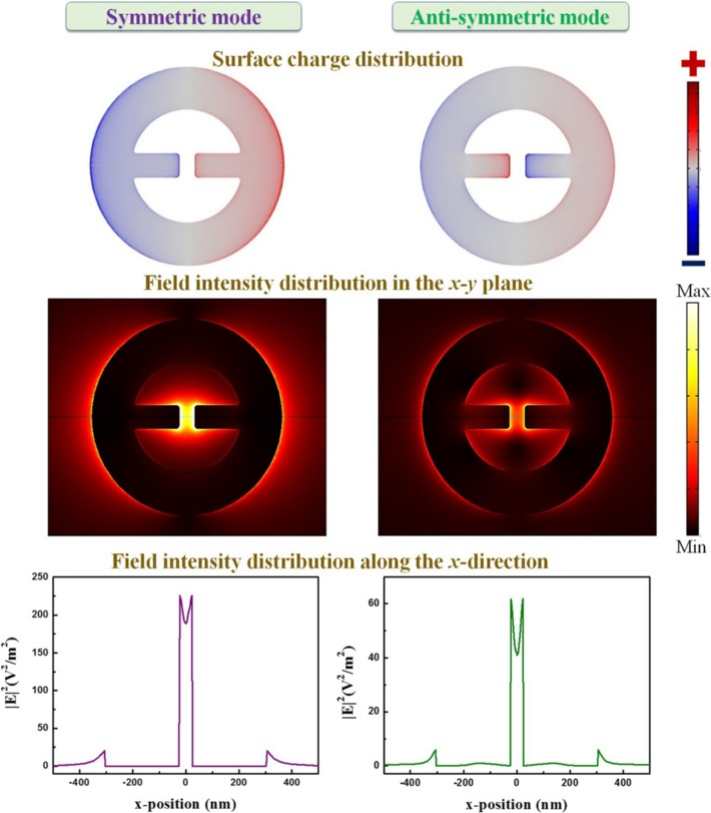
Surface charge and field intensity distributions of the symmetric and anti-symmetric modes in the *x*-*y* plane and along the *x*-direction

To identify the peaks, the corresponding surface charge distributions are calculated, and these results are shown in the upper figure of Fig. 2. From the symmetry characteristic of the surface charge distributions on the ring and rod dimer in MINE, we name the longer wavelength peak (purple arrow) symmetric mode and the shorter-wavelength peak (green arrow) anti-symmetric mode. Other peaks in the even shorter wavelength region are identified as high-order modes (black arrows in Fig. 1c). In addition, the unapparent shoulder in the spectral range between 950 and 1050 nm in the measured spectrum can be ascribed to the grating-induced mode that occurs in the grating or array structure [38, 39]. The symmetric and anti-symmetric modes are a focus of the following discussion because they can be used to achieve high local near-field enhancement in the MINE structure. The corresponding electric-field intensity distributions of the two modes in the *x*-*y* plane (*z* = 15 nm) and along the *x*-direction (*y* = 0 nm, *z* = 15 nm) are provided in the middle and lower figures of Fig. 2, respectively. Compared to the anti-symmetric mode, the symmetric mode enables a stronger near field with a concentrated distribution around the gap. Obviously, the field intensity of the symmetric mode in the gap region is stronger than that of the anti-symmetric mode. This occurs because the cavity field induced by the ring and the gap field of the rod dimer in the symmetric mode are in the same direction, which makes the coupling stronger and results in a much enhanced near field around the gap. On the other hand, the coupling for the anti-symmetric mode is weaker owing to the opposite directions of the established fields in the cavity and the gap. In addition, the symmetric and anti-symmetric modes correspond to dipole and quadrupole modes. This also clarifies the superior field enhancement by a bright dipole mode compared to a dark quadrupole mode. Therefore, the field intensity of the symmetric mode at the gap center can be enhanced by a factor of ~190, while the enhancement factor of the anti-symmetric mode is only ~40.

**Fig. 3:**
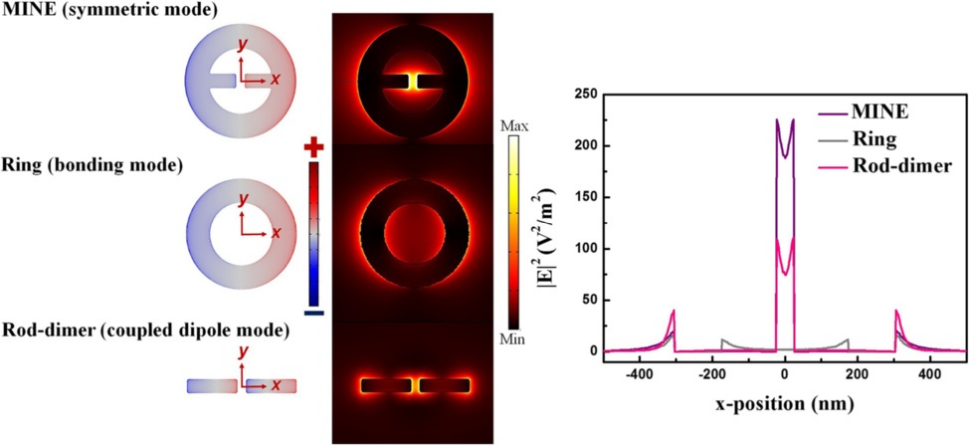
Surface charge and field intensity distributions in the *x*-*y* plane and field intensity distributions along the *x*-direction of the MINE, nanoring, and nanorod dimer structures

For comparison, the surface charge and field intensity distributions in the *x*-*y* plane and field intensity distributions along the *x*-direction of the MINE (symmetric mode), nanoring with a thickness of 30 nm, inner radius of 175 nm, and outer radius of 305 nm (bonding mode) and nanorod dimer with a thickness of 30 nm, gap of 50 nm, rod width of 75 nm, and rod length of 280 nm (coupled dipole mode) structures are shown in Fig. 3. Note that these distributions are calculated at their own resonance wavelengths. The profile of the enhanced field of the MINE structure can be viewed as a superposition of those of the nanoring and nanorod dimer structures, but its field intensity at the gap is much higher than that of the nanoring or nanorod dimer. The field enhancement at the outer edges of the MINE structure is smaller than that of the nanorod dimer since the auxiliary nanoring reduce the charge densities at the outer edges. The finding is in agreement with ref. [40]. It is well-known that the nanorod dimer is able to gain a high local field at the center of the structure by adjusting its rod width, rod length, and gap distance. When adopting an auxiliary nanoring structure surrounding the nanorod dimer to form the MINE structure, the local field can be further enhanced owing to the excess charges from the nanoring. As shown in Fig. 3, the highest field intensity of ~190 V^2^/m^2^ at the gap center for the MINE structure is 2.5 times larger than that for the nanorod dimer. From the viewpoint of the nanoring, its plasmon resonance is highly tunable through the variations of its inner and outer radii. By adopting a nanorod dimer within the nanoring to form the MINE structure, the rod serves as a bridge to pull the charges from the ring to rod, which induces strong plasmon coupling between the rods and results in high local near-field enhancement. The maximum near-field intensity of the MINE structure is over ten times higher than that of the nanoring structure. These results show that our proposed MINE structure enables significant enhancement on the local near field compared to the individual nanorod dimer or nanoring. Based on this concept, one can properly add an auxiliary ring-shaped structure to any dimer system (e.g., bowtie structure) to efficiently improve the localized and enhanced near-field intensity inside the gap of the dimer system. In a fan-rod electric antenna and a spiky nanoparticle dimer, the flared section and the bulky spherical core work as electron reservoirs similar to the auxiliary nanoring in this study [40, 41]. The reservoir can supply a large amount of charges into the sharp dimer structure which can confine the accumulation of charges to apexes. Thus, the better synergistic interaction of “plasmon coupling” and “lightning-rod effect” can be achieved for high-field enhancement around the nanoscale gap. A single cross-junction ring antenna is also similar to the MINE structure, and it can support a high-field intensity at a cross junction because a field can be further concentrated at the center of the structure via attaching a ring to a cross rod dimer [42].

### Influences of Gap, Outer Radius, and Rod Width on Plasmonic Properties

**Fig. 4:**
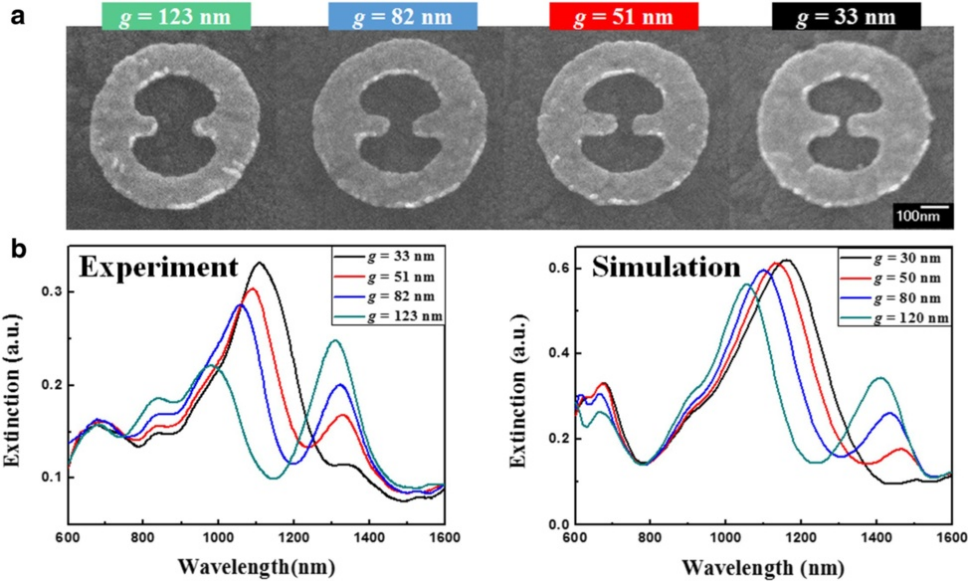
**a** SEM images of the fabricated MINE structures with different gap distances. **b** Extinction spectra of the MINE structures with different gap distances for both experiment and simulation

**Fig. 5:**
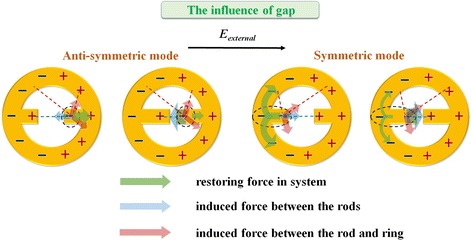
Schematic illustrations of the instantaneous charge distributions in MINE structures: the influences of gap

**Fig. 6:**
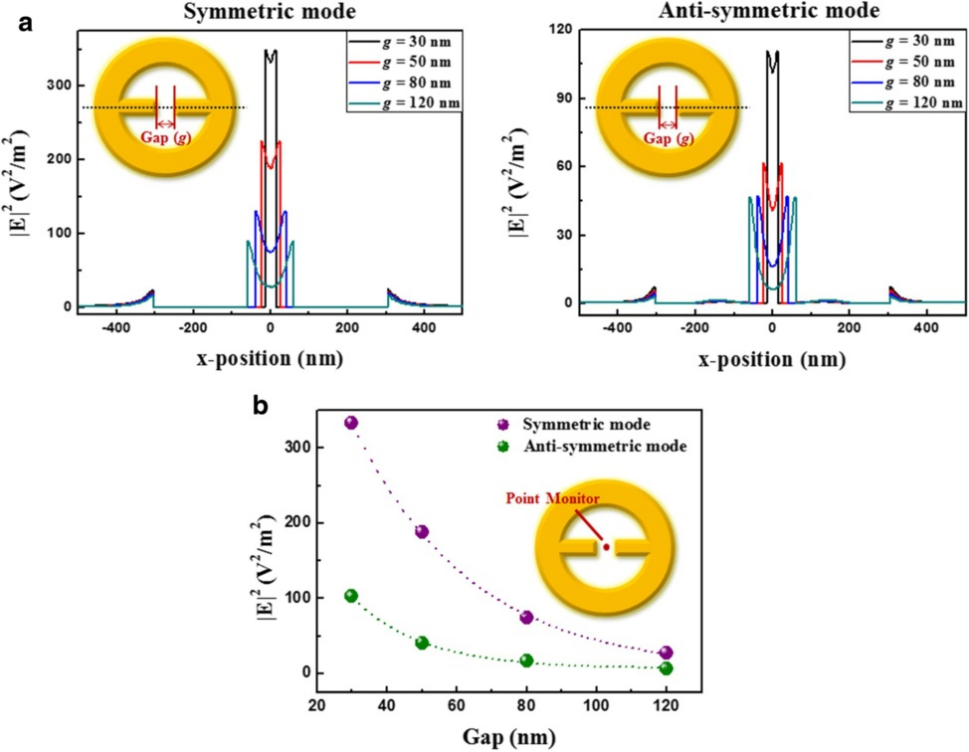
**a** Field intensity distributions of the symmetric and anti-symmetric modes of the MINE structures along the *x*-direction with different gap distances. **b** Field intensities at the gap center of the MINE structure as a function of the gap distance

**Fig. 7:**
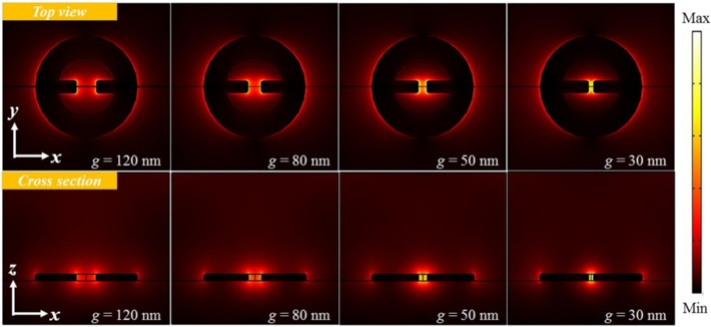
Field intensity distributions of the symmetric mode of MINE structures in the *x*-*y* and *x*-*z* planes with different gap distances

To investigate the influence of gap on the plasmonic properties of MINE structures, a series of MINE arrays are fabricated with various gap distances from 33 to 123 nm and fixed *t* = 31 nm, *w* = 77 nm, *r*_*i*_ = 174 nm, and *r*_*o*_ = 304 nm. The corresponding SEM images are shown in Fig. 4a. The measured and simulated extinction spectra are provided in Fig. 4b. The experimental results closely agree with the simulated ones, showing the same trends of relative peaks and amplitudes when varying the gap distance. Since the anti-symmetric mode is more like a dark mode (quadrupole mode), its intensity should be lower than that of the symmetric mode (bright dipole mode). However, this is not observed because the free carrier absorption becomes stronger as the wavelength increases in the NIR region for the ITO glass substrate used in simulations and experiments [37]. Therefore, scattered light caused by the dipole-like symmetric mode is absorbed by the ITO glass substrate, resulting in the reduced intensity of the symmetric mode in the extinction spectra. Moreover, the resonance peaks of the symmetric and anti-symmetric modes reveal a considerable red shift when the gap distance is reduced. This shift can be explained from the viewpoint of the restoring force in the nanostructure or coupling system [43, 44]. Figure 5 shows schematic illustrations of the instantaneous charge distributions for the symmetric and anti-symmetric modes of the MINE structure under the influence of gap. The restoring force (semitransparent green arrow) induced attractive force between the rods (semitransparent blue arrow) and induced attractive and repulsive forces between the rod and ring (semitransparent magenta arrow) for negative charges (black dashed circle) are marked in these schematic illustrations. With the reduced gap distance, the induced forces between the rod and ring are only changed slightly, but the stronger coupling effect will induce a larger attractive force in the gap region, thereby weakening the restoring force in the structure. Furthermore, the field intensity distributions of the two modes along the *x*-direction are shown in Fig. 6a. For the symmetric and anti-symmetric modes, the field intensities in the gap can be significantly enhanced with the decrease of the gap distance. The field intensity at the center of the MINE structure as a function of gap distance is provided in Fig. 6b. When a nanorod dimer is adopted within the nanoring, the MINE structure exhibits an exponential field enhancement when the gap becomes smaller, which echoes the observations in many dimer systems [45–47]. The remarkable enhancement of the near field is a consequence of the strengthened plasmon coupling between the rods [2, 48]. When the gap distance is decreased from 120 to 30 nm, the intensity enhancement of around 12.5 times is obtained for the symmetric mode. Moreover, the field intensity of the symmetric mode at the gap center is 3.4 times stronger than that of the anti-symmetric mode when *g* = 30 nm. This again is because the cavity field of the ring and the gap field of the rod dimer in the symmetric mode are in the same direction. For the symmetric mode, field intensity distributions in the *x*-*y* and *x*-*z* planes are shown under different gap distances in Fig. 7.

When the gap distance reduces from 120 to 30 nm, a greatly enhanced and strong near field is induced in the gap region, and the plasmonic mode is gradually localized to an extremely small range as well. Such significantly enhanced and localized near-field property dominates the optical interaction with absorbed biomolecules or with fluorophores passing in close proximity, which is helpful for the improvement of spectroscopic signal and fluorescent efficiency. Therefore, this novel MINE structure has great potential for use in practical applications.

**Fig. 8:**
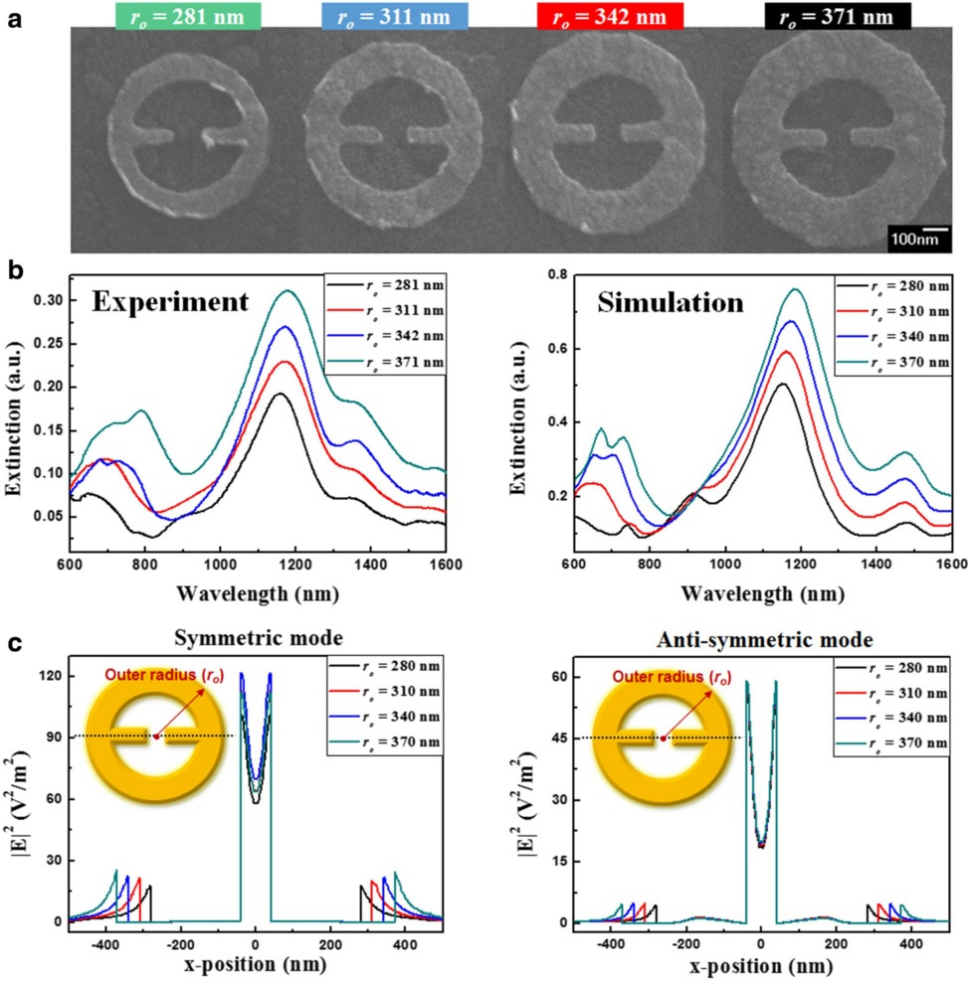
**a** SEM images of the fabricated MINE structures with different outer radii. **b** Extinction spectra of the MINE structures with different outer radii for both experiment and simulation. **c** Field intensity distributions of the symmetric and anti-symmetric modes of MINE structures along the *x*-direction with different outer radii

**Fig. 9:**
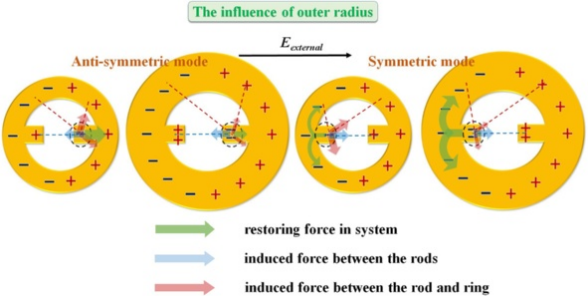
Schematic illustrations of the instantaneous charge distributions in MINE structures: the influences of outer radius

For the influence of outer radius, MINE arrays are fabricated with various outer radii from 281 to 371 nm and fixed *t* = 31 nm, *w =* 66 nm, *g* = 83 nm, and *r*_*i*_ = 203 nm. The corresponding SEM images are shown in Fig. 8a. The measured and simulated extinction spectra are provided in Fig. 8b. The resonance peaks of symmetric and anti-symmetric modes reveal slight blue and red shifts, respectively, when the outer radius is enlarged. For the symmetric (anti-symmetric) mode, the restoring force becomes stronger (weaker) with the increase in the outer radius because the attractive force in the gap region only varies a little but the induced forces between the rod and ring are decreased, as shown in Fig. 9. In addition, the extinction ability can be increased for the relatively larger outer radius for both modes. This phenomenon could occur because the large structural size supports more charges for inducing a larger net dipole moment, thereby promoting the extinction ability. The field intensity distributions of symmetric and anti-symmetric modes along the *x*-direction under different outer radii are plotted in Fig. 8c. The fields in the gap remain nearly the same as the outer radius is varied, which implies that the outer radius variation has little impact on the near-field distribution and is not a dominant factor for near-field enhancement. Therefore, adopting a nanoring surrounding the nanorod dimer, by enlarging its outer radius, more charges can be induced in the MINE structure and the extinction ability can be increased, as observed in Fig. 8b. This feature is also beneficial for practical applications.

**Fig. 10:**
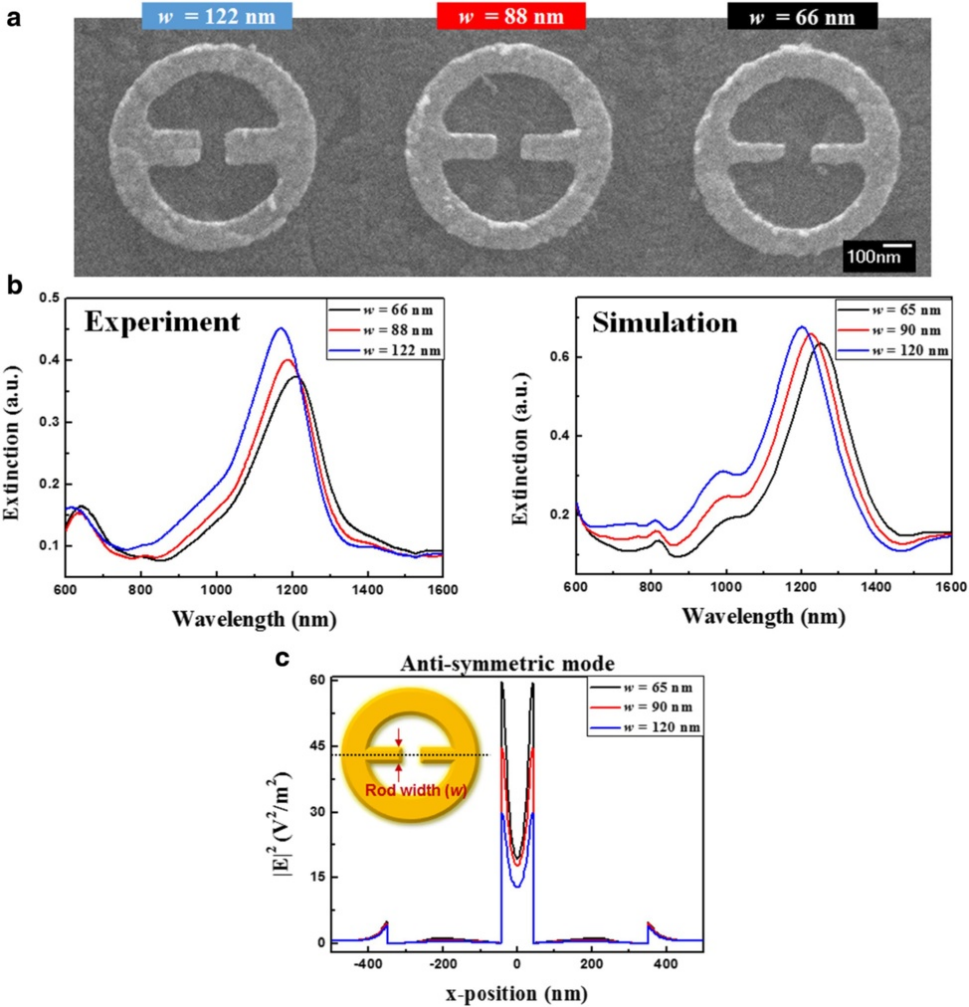
**a** SEM images of the fabricated MINE structures with different rod widths. **b** Extinction spectra of the MINE structures with different rod widths for both experiment and simulation. **c** Field intensity distributions of the anti-symmetric modes of MINE structures along the *x*-direction with different rod widths

**Fig. 11:**
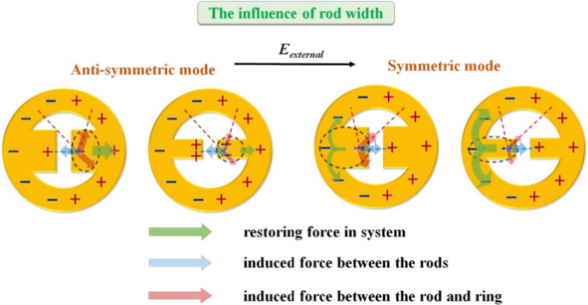
Schematic illustrations of the instantaneous charge distributions in MINE structures: the influences of rod width

To investigate the influence of rod width, MINE arrays are fabricated with various rod widths from 66 to 122 nm and fixed *t* = 31 nm, *g =* 84 nm, *r*_*i*_ = 252 nm, and *r*_*o*_ = 349 nm. The corresponding SEM images are shown in Fig. 10a. The measured and simulated extinction spectra are provided in Fig. 10b. For the anti-symmetric mode, the resonance peak red shifts to long wavelength when the rod width decreases. With the reduced rod width, the attractive force between the rods increases slightly but the forces induced between the rod and ring decreases significantly, which results in weakened restoring force, thereby shifting the resonance peak to long wavelength, as shown in Fig. 11. However, the symmetric modes are barely observable as a result of strong free carrier absorption of ITO in the NIR region. When the wavelength becomes longer, the increased absorption weakens the extinction ability of the MINE structure, making the symmetric mode nearly invisible. This can be improved by using substrates without absorption (e.g., glass, quartz, and CaF_2_) in the applied wavelength range. Even though it is difficult to observe the shift from unapparent symmetric mode, we anticipate that a blue shift should occur owing to the strengthened restoring force in system because the induced forces between the rod and ring become smaller. The field intensity distributions of the anti-symmetric mode along the *x*-direction under different rod widths are plotted in Fig. 10c. Clearly, when the rod width is decreased from 120 to 65 nm, the field intensity at the gap center can be further increased. Moreover, a much more significant enhancement on the field intensity at the rod tips is observed. This can be attributed to the more intensive lightning effect owing to its sharper shape and makes the MINE structure suitable for optical trapping that needs large field gradient.

**Fig. 12:**
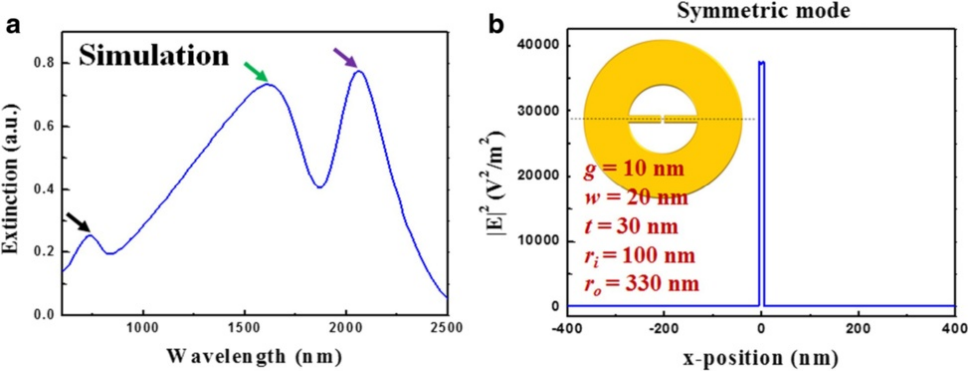
**a** Extinction spectrum and **b** field intensity distribution of the symmetric mode along the *x*-direction of the MINE structure with *g* = 10 nm, *w* = 20 nm, *t* = 30 nm, *r*_*i*_ = 100 nm, and *r*_*o*_ = 330 nm on glass substrate

In summary, the optical properties of the MINE structure highly depend on the structural geometry. The influences of the gap, outer radius, and rod width are systematically investigated. To boost the performance with respect to the highly localized and enhanced field and increase the extinction ability of the MINE structure, the gap, outer radius, and rod width should be as smaller, larger, and narrower as possible owing to the better plasmon coupling, more induced charges, and stronger lightning-rod effect. Besides, substrates without absorption in the applied wavelength range can be utilized to avoid the degraded extinction. For a MINE structure with *g* = 10 nm, *w* = 20 nm, *t* = 30 nm, *r*_*i*_ = 100 nm, and *r*_*o*_ = 330 nm on a glass substrate, the simulated extinction spectrum and field intensity distribution of the symmetric mode along the *x*-direction are shown in Figs. 12a, b. The near-field intensity at the gap center can be dramatically enhanced by a factor of ~3.72 × 10^4^ for the symmetric mode with good extinction ability.

## Conclusions

A novel MINE structure, which has the combined features of the nanorod dimer and nanoring, is proposed and fabricated, and its plasmonic behaviors are investigated numerically and experimentally. Two resonance peaks are identified as the symmetric and anti-symmetric modes according to the symmetries of the charge distributions on the nanoring and nanorod dimer in MINE. The symmetric mode is preferred because its charge distribution leads to a stronger near field with a concentrated distribution around the gap. In addition, the optical properties of the MINE structure highly depend on the structural geometry. The near-field intensity can be greatly enhanced by adjusting the rod width and gap distance, promoting the lightning-rod effect and plasmon coupling. The extinction ability can be raised by enlarging the outer radius because more charges are supported by the larger structural size. Based on the strong and localized near field at the center of nanorod dimer, our studies show that adopting an auxiliary nanoring surrounding the nanorod dimer can further enhance its near field. Compared to individual nanoring and nanorod dimer, our proposed MINE structure can dramatically boost the local near-field intensity because excess charges can be induced by the ring-shaped nanostructure and the rod-shaped nanostructure can function as a bridge to pull these excess charges from the ring to the rods, thereby inducing stronger plasmon coupling between the rods and resulting considerably local near-field enhancement. Hence, this MINE structure, which has a significantly enhanced and localized near field, has great potential for applications such as improving single-molecule detection and exploring biochemistry, including molecular bonding and chemical reactions. Our studies provide illuminating insight into complex systems and guide the design of an optimal MINE structure. Moreover, we expect that this nanostructure can serve as a building block for various applications such as nanomedicine, biochemistry, single-emitter fluorescence, vibrational spectroscopy, and optical tweezers.
